# High DHCR7 Expression Predicts Poor Prognosis for Cervical Cancer

**DOI:** 10.1155/2022/8383885

**Published:** 2022-09-16

**Authors:** Jing Zou, Sha Liu, Jian Long, Bingbing Yan

**Affiliations:** ^1^Department of Oncology, Jingzhou Hospital, Yangtze University, Hubei 434020, China; ^2^Department of Gynecology, The Maternal and Child Health Hospital of Guangxi Zhuang Autonomous Region, Guangxi, China

## Abstract

DHCR7 is a rate-limiting enzyme in cholesterol synthesis. The expression pattern and prognostic value of DHCR7 in cervical cancer are unknown. We investigated the relationship between DHCR7 expression and clinicopathological features of cervical cancer patients. The dataset was acquired from TCGA database. The Wilcoxon rank sum test was used to explore DHCR7 expression level in cervical cancer. The Kruskal-Wallis test and the logistic regression were performed to estimate the association between the DHCR7 and clinical features. The Kaplan-Meier and Cox regression analyses were used to evaluate factors that affect cervical cancer prognosis. GSEA was used to screen the DHCR7-related pathways. We found that DHCR7 was increased in cervical cancer samples and increased DHCR7 was correlated with advanced T stage, lymph node invasion, and clinical stage (*P* < 0.05). Patients with elevated DHCR7 levels had poorer overall survival (*P* = 0.021), progression-free interval (*P* = 0.002), and disease-specific survival (*P* = 0.005). Cox analysis revealed that DHCR7 was an independent prognostic factor in cervical cancer (*P* = 0.005). WNT activated receptor activity, G2/M checkpoint, mTORC1 signaling, KRAS signaling, regulation of cholesterol biosynthetic, FGF signaling, T-cell receptor signaling, JAK/STAT signaling cascade T cell activation, and macrophage migration were enriched in high DHCR7 phenotype. Our data also showed that DHCR7 moderately correlates with T-cell infiltration, including CD8^+^ T-cells. *Conclusion.* Increased DHCR7 expression is associated with poor survival in cervical cancer.

## 1. Background

Cervical cancer is one of the most common cancers worldwide and is associated with high mortality rate. It is a major health issue for women, especially in developing countries [1]. Surgery, radiotherapy, and chemotherapy are the main treatment strategies for cervical cancer [2]. Although the survival of cervical cancer has been improved with the combination of the above treatments, the prognosis of high-risk patients remains poor, especially those with metastasis and recurrent diseases [3]. Discovery of biomarkers for diagnosis and therapy may help to improve the prognosis of cervical cancer.

DHCR7 (7-dehydrocholesterol reductase) is a rate-limiting enzyme in cholesterol synthesis, which is mainly involved in the conversion of 7-dehydrocholesterol (7-DHC) to cholesterol [4]. It has been pointed out that cholesterol has an impact on the prognosis of several types of cancers, including cervical cancer [5]. DHCR7 deficiency causes Smith-Lemli-Opitz syndrome (SLOS), which is an autosomal recessive syndrome charactered by multiple congenital abnormalities, including morphogenic, mental retardation and behavioral disorders, due to lack of cholesterol-derived steroid hormones [6]. DHCR7 is also considered to be a vitamin D-related gene which may have an influence on calcium homeostasis, bone health, and various cancers [7–9]. Carvalho et al. [10] showed that DHCR7 polymorphisms might increase the risk of thyroid cancer via its effects on circulating vitamin D levels. However, there is no report about the prognostic value of DHCR7 in cervical cancer.

Here, we used the RNA-Seq data and clinical information from TCGA database to explore the prognostic value of DHCR7 in cervical cancer. Firstly, we compared the DHCR7 expression between cervical cancer tissues and normal tissues. Then, we studied the relationship between DHCR7 expression and clinicopathological characteristics. Cervical cancer patients were clustered into 2 groups based on the DHCR7 expression level. The Kaplan-Meier method and Cox regression analyses were then utilized to compare the survival of patients with different DHCR7 expression levels. Gene ontology (GO) and KEGG enrichment analyses were used to explore the biological role of DHCR7. Furthermore, DHCR7-related biological pathways involved in cervical cancer were screened by gene set enrichment analysis (GSEA). Additionally, we also explored its role in tumor immunity.

## 2. Materials and Methods

### 2.1. RNA Sequencing Data Source and Data Preprocessing

RNA sequencing data were obtained from TCGA (uhttps://http://portal.gdc.cancer.gov/) (normal cervical tissues and cervical cancer tissues) and GTEx (normal cervical tissues). RNA-seq data were analyzed to determine DHCR7 expression level via UCSC's Xena (https://xena.ucsc.edu/). UCSC's Toil algorithm [11] was used to diminish the batch effects. Patients with incomplete clinical information or gene expression data were excluded from the analysis. Since the present study met the publication guidelines stated by TCGA (https://cancergenome.nih.gov/publications/publicationguidelines) and the data were obtained from TCGA, ethical approval and informed consent were not required.

### 2.2. Identification of Differentially Expressed Genes (DEGs)

Tumor samples were grouped into DHCR7-high and DHCR7-low expression groups based on the median DHCR7 expression level. The expression profiles of DHCR7-high and DHCR7-low expression groups were compared using DESeq2 (3.8) package of R [12]. Genes with |log2 fold change (FC)| >1 and adjust (adj.) *P*-value <0.05 were considered DEGs. Gene expression differences were visualized using volcano plot and heat map.

### 2.3. Functional Enrichment Analysis

Gene ontology (GO) [13] functional analysis and KEGG [14] pathway analysis were performed using the clusterProfiler method (3.14.3) [15]. FDR (false discovery rate) <0.05 and adj. *P*-value <0.05 were considered statistically significant.

### 2.4. Gene Set Enrichment Analysis (GSEA)

Gene set enrichment analysis (GSEA) [16] of DHCR7-high and DHCR7-low groups were conducted using the clusterProfiler package of R software. The analysis involved 1000 permutations for each analysis. Adj. *P*-value <0.05, FDR *q*-value <0.25, and normalized enrichment score (|NES|) ≥1 were employed to identify enriched pathways.

### 2.5. Inference of Infiltrating Immune Analysis

Immunocyte signatures involving 509 genes were used to predict the infiltration level of 24 immune cells in each tissue sample [17]. Immune infiltration level of immune cells was analyzed via single-sample gene set enrichment analysis (ssGSEA) [18] using GSVA (http://bioconductor.org/packages/release/bioc/html/GSVA.html) package on R. To assess the correlation between DHCR7 expression and immune cells infiltration levels, Spearman's correlation analysis was performed. Immune cell infiltration in DHCR7-high and DHCR7-low expression groups was compared using the Wilcoxon rank sum test.

### 2.6. Protein-Protein Interaction (PPI) Network

To further study the functional interaction between DEGs, Metascape [19] online analysis (http://metascape.org) (update date: 2020-3-20) was used to construct a PPI network of the DEGs. Terms with *P* < 0.01, a minimum count of 3, and the enrichment factor >1.5 were regarded as significant. Hub genes and significant modules of this PPI network were extracted by Molecular Complex Detection (MCODE) [20] algorithm.

### 2.7. Detection of DHCR7 Expression by Western Blotting

Cervical cancer cells were cultured in RPMI 1640 medium (Gibco, Thermo Fisher Scientific, Inc., MA, USA) supplemented with 10% fetal bovine serum (FBS) (Gibco, Thermo Fisher Scientific, Inc., MA, USA). Cells were incubated at 37°C in a 5% CO2 humidified incubator. Protein lysis buffer (Beyotime, Shanghai, China) was used to extract the cell lysates and bicinchoninic acid (BCA) protein assay kit (Beyotime, Shanghai, China) was used to determine the protein concentration. The protein samples were resolved by SDS-PAGE and then transferred to a polyvinylidene fluoride membrane (EMD Millipore, MA, USA). 5% bovine serum albumin (Beyotime, Shanghai, China) was used to block the membranes. Rabbit anti-DHCR7 antibody (1 : 2000, Invitrogen) and rabbit anti-*β*-actin were used to react with the protein samples at 4°C overnight. Then, goat anti-rabbit antibody (1 : 1000, Beyotime, Shanghai, China) was used to incubate the membranes at room temperature for 1 h. ECL plus reagent (Beyotime, Shanghai, China) was used to detect the brands.

### 2.8. Downregulation of DHCR7 Expression

About 2 × 10^5^ cells were seeded into each well of the 6-well plates and incubated at 37°C in a 5% CO2 humidified incubator overnight so that the cells were adherent to the plates. 100 pmol of siRNA of DHCR7 (si-DHCR7) or negative control and 7.5uL lipo3000 transfection reagent (Invitrogen) were added to each well and the cells were incubated for 48 h. Then, DHCR7 expression was detected by western blotting. Sequences of si-DHCR7: sense: 5′- CUAUAUGAUGGGAAUUGAG -3′, antisense: 5′- GAUAUACUACCCUUAACUC -3′.

### 2.9. Cell Proliferation Was Determined by CCK8 Assay

Cells were cultured in RPMI 1640 medium supplemented with 10% FBS by 96-well plates for 24 h, 48 h, and 72 h, respectively. Then, the medium was replaced by RPMI 1640 medium not containing serum and 20*μ*l CCK8 reagent (Beyotime, Shanghai, China) was added to each well and incubated in dark for 2 h. The optical density (OD) at 490 nm was detected.

### 2.10. Statistical Analysis

Statistical analyses were performed using R software (v3.6.2). The Wilcoxon rank sum test was employed to evaluate DHCR7 expression in 304 cervical cancer and 13 normal samples. DHCR7 protein expression in normal vs. cervical cancer tissues was assessed using immunohistochemistry (IHC) images on Human Protein Atlas (HPA, https://www.proteinatlas.org). The “pROC” package on R was used for ROC (receiver operating characteristic) analysis to determine the diagnostic power of DHCR7 in cervical cancer [21]. Relationship between DHCR7 expression and clinicopathological features was tested using the Kruskal-Wallis test, Wilcoxon signed-rank test, and logistic regression analysis. Survival was analyzed using the Kaplan-Meier method and tested by log-rank test. Univariate and multivariate Cox regression analyses were used to analyze the impact of different characteristics on patient prognosis. Overall survival (OS), progression-free interval (PFI), and disease-specific survival (DSS) were used to evaluate the clinical outcomes. The HR (95% confidence interval; CI) was used to evaluate hazard risk for individual factors. A nomogram was used to predict patient prognosis using rms package on R based on multivariate analysis results. Calibration plots were used to assess the nomogram's prediction accuracy. *P* < 0.05 indicated statistically significant differences.

## 3. Results

### 3.1. Clinical Characteristics

Toil was used to unify TPM format of RNA-seq data from 317 samples. 304 samples were cervical cancer samples from TCGA and 13 samples were normal tissues from TCGA and GTEx. Patient characteristics, including age, metastasis (M stage), lymph node status (N stage), clinical stage, T stage (primary tumors), histological type, histological grade, radiation therapy, menopause status, primary therapy outcome, birth control pill history, and smoking history, were recorded. Median age at diagnosis was 46 years old. 52 (17.1%) adenosquamous cases and 252 (82.9%) squamous cell carcinoma cases were included. There were 162 (53.2%), 69 (22.6%), 45 (14.8%), and 21 (6.9%) cases of patients with stages I, II, III, and IV diseases, respectively. Sixty (19.7%) patients had lymph node invasion (N1) and 10 (3.3%) patients had distant metastasis (M1) (Table 1).

### 3.2. DHCR7 Expression and Clinicopathological Characteristics

**Figure 1:**
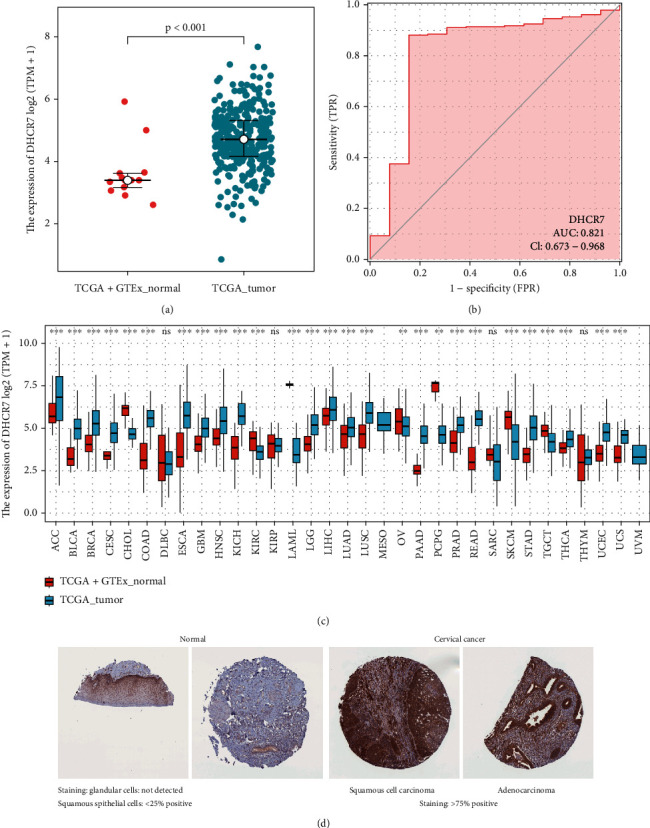
DHCR7 expression in tumor and normal tissues. (a) DHCR7 mRNA expression in cervical cancer samples and normal cervical samples. (b) The ROC curve of DHCR7 in cervical cancer. (c) Expression of DHCR7 in 33 types of cancers and non-cancer samples. ns, *P* ≥ 0.05. ^∗^, *P* < 0.05. ^∗∗^, *P* < 0.01. ^∗∗∗^, *P* < 0.001. (d) Representative immunohistochemistry images of DHCR7 expression in cervical cancer tissues (intensitive, strong. Quantity, >75% positive) and normal tissues (intensitive, weak. Quantity, <25% positive) (Human Protein Atlas). DHCR7 protein expression was weak in non-tumor tissues and strong in cervical cancer tissues.

**Figure 2:**
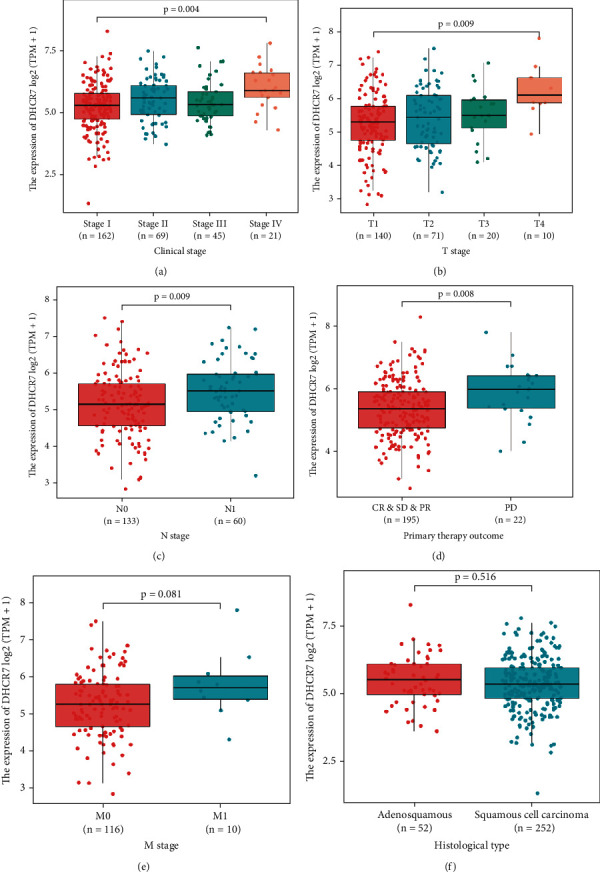
Association between DHCR7 expression and clinicopathologic characteristics. (a) Clinical stage. (b) T stage (primary tumor). (c) N stage (lympho node invasion status). (d) Primary therapy outcome. (e) M stage (metastasis). (f) Histological type. High DHCR7 expression was correlated with poor clinicopathological features.

The Wilcoxon rank sum test analysis of DHCR7 expression levels in cervical cancer samples vs. normal samples revealed that DHCR7 level was significantly higher in cervical cancer tissues (*P* < 0.001; Figure 1(a)). Assessment of DHCR7 protein levels on Human Protein Atlas showed that expression intensity of DHCR7 was weak in non-tumor cervical tissues and strong in cervical cancer tissues (Figure 1(d)). To obtain a more comprehensive evaluation of DHCR7 expression in cancer, we compared DHCR7 mRNA expression in the TCGA pan-cancer cohort which included 33 types of cancers. Data analysis showed that DHCR7 expression was elevated in 27 types of cancer types in comparison with normal samples (Figure 1(c)). ROC curve analysis indicated that DHCR7 expression efficiently discriminated between tumor and non-tumor cervical tissues, with an area under the ROC curve (AUC) of 0.821 (95% CI: 0.673-0.968) (Figure 1(b)). The ROC curve suggested that DHCR7 had strong diagnostic power for cervical cancer. Comparison of DHCR7 expression in cervical cancer patients with different clinicopathological features revealed that DHCR7 expression increased with advanced clinical stage (*P* = 0.004) and T stage (primary tumors) (*P* = 0.009). DHCR7 was also upregulated in patients with lymph nodes invasion (N1) (*P* = 0.009). Patients with worsen primary therapy outcome (PD) also showed higher DHCR7 expression level (*P* = 0.008) (Figures 2(a)–2(d)). However, DHCR7 expression showed no difference in patients with different metastatic status (M stage) or different histological types (*P* > 0.05) (Figures 2(e) and 2(f)). Logistic regression analysis revealed that high DHCR7 levels were correlated with advanced T stage (OR =1.01 (1.00-1.02), *P* = 0.028), lymph node invasion (OR =1.01 (1.00-1.02), *P* = 0.043), metastasis (OR =1.01 (1.00-1.03), *P* = 0.049), advance clinical stage (OR =1.01 (1.00-1.01) for stage II and stage III and stage IV vs. stage I, *P* = 0.026), and primary therapy outcome (OR =1.01 (1.00-1.01) for PR and SD and PD vs. CR, *P* = 0.012) (Table 2).

### 3.3. Survival Outcomes and Multivariate Analysis

**Figure 3:**
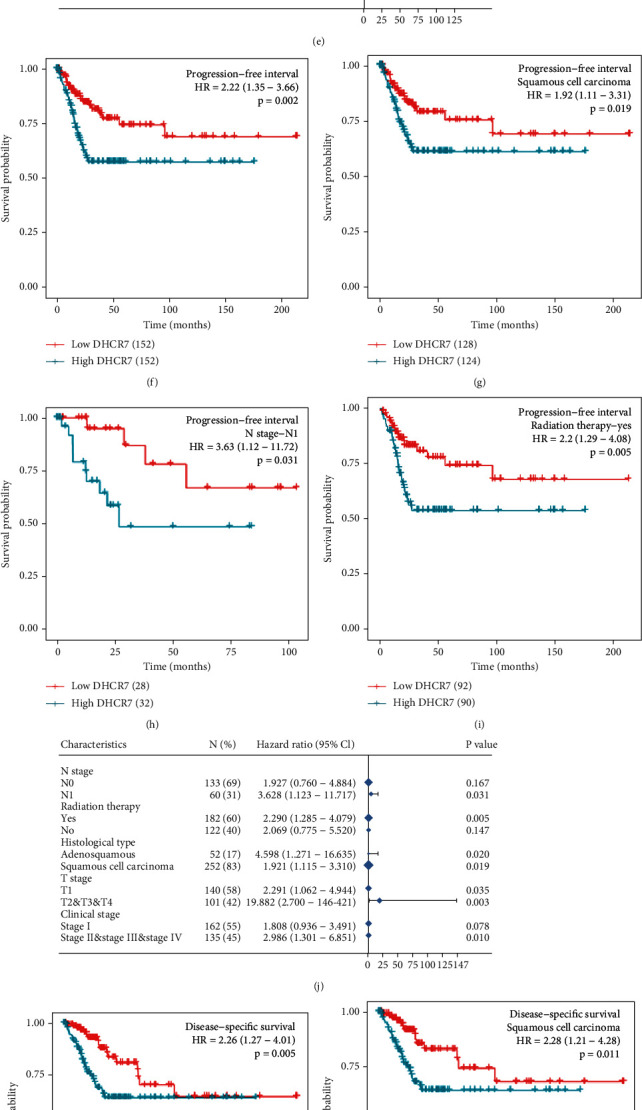
The prognostic value of DHCR7 for cervical cancer. (a) Kaplan-Meier analysis of OS in low- and high-DHCR7 groups. (b–d) OS in clinicopathological subgroups of squamous cell carcinoma, lymph node metastasis, and radiation therapy. (e) The hazard ratio of different clinicopathological features to OS in different clinicopathological subgroups. (f) Kaplan-Meier analysis of PFI in low- and high-DHCR7 groups. (g–i) PFI in clinicopathological subgroups of squamous cell carcinoma, lymph node metastasis, and radiation therapy. (j) The hazard ratio of different clinicopathological features to PFI in different clinicopathological subgroups. (K) Kaplan-Meier analysis of DSS in low- and high-DHCR7 groups. (l–n) DSS in clinicopathological subgroups of squamous cell carcinoma, lymph node metastasis, and radiation therapy. (o) The hazard ratio of different clinicopathological features to DSS in different clinicopathological subgroups. OS: overall survival; DSS: disease-specific survival; PFI: progression-free interval; DFI: disease-free interval.

304 cervical cancer cases with clinical outcome information were grouped into DHCR7-low and DHCR7-high groups based on the median DHCR7 expression level. The Kaplan-Meier analyses showed that the OS (*P* =0.021), PFI (*P* =0.002), and DSS (*P* = 0.005) (Figures 3(a), 3(f), and 3(k)) of patients in DHCR7-low group were significantly better than patients in DHCR7-high group. We further conducted a subgroup analysis in patients with different clinical characteristics. In squamous cell carcinoma subgroup, patients with high DHCR7 expression showed obviously worse OS (*P* = 0.025), PFI (*P* = 0.019), and DSS (*P* = 0.011) (Figures 3(b), 3(g), and 3(l)). In adenosquamous subgroup, patients with high DHCR7 also showed shorter PFI (*P* = 0.02) (Figure S1B). However, there were no OS or DSS differences between patients with different DHCR7 expression levels (Figure S1A, C). And we also found that in lymph nodes invasion subgroup (N1), high DHCR7 patients had worse OS, PFI, and DSS than low DHCR7 patients (*P* < 0.05) (Figures 3(c), 3(h), and 3(m)). In patients who received radiation therapy, OS, PFI, and DSS were significantly shorter for high-DHCR7 group (*P* < 0.05) (Figures 3(d), 3(i), and 3(n)). The hazard ratio of different clinicopathological features to OS, PFI, and DSS was showed in the forest plots (Figures 3(e), 3(j), and 3(o)).

Univariate Cox regression analysis showed that high-DHCR7 patients had worse OS (HR 1.758 (1.088-2.841), *P* = 0.021), PFI (HR 2.224 (1.352-3.657), *P* = 0.002), and DSS (HR = 2.258 (1.271-4.013), *P* = 0.005) than low-DHCR7 patients (Table 3(a)). Multivariate Cox regression analysis was further performed to investigate the independent prognostic factors for cervical cancer. It was indicated that high DHCR7 expression level was independently associated with unfavorable OS, PFI, and DSS (*P* < 0.05) (Table 3(b)). DHCR7 and other independent clinical risk factors (primary therapy outcome and lymph nodes (N)) were included in the nomogram analysis, which was aimed to predict cervical cancer prognosis. The C-index value was 0.760 (95% CI, 0.698-0.822) for OS, 0.703 (95% CI, 0.645-0.760) for PFI, and 0.802 (95% CI, 0.751-0.852) for DSS (Figure S2A, C, E). Calibration curve analysis presented good agreement between fit and actual observation for 1-, 3-, and 5-year OS, PFI, and DSS (Figure S2B, D, F). These results suggested that DHCR7 expression level effectively predicted the OS, FPI, and DSS for cervical squamous cell carcinoma patients, with reliable performance. High DHCR7 levels may be a biomarker of poor prognosis for cervical cancer.

### 3.4. Identifying Differentially Expressed Genes in Cervical Cancer

**Figure 4:**
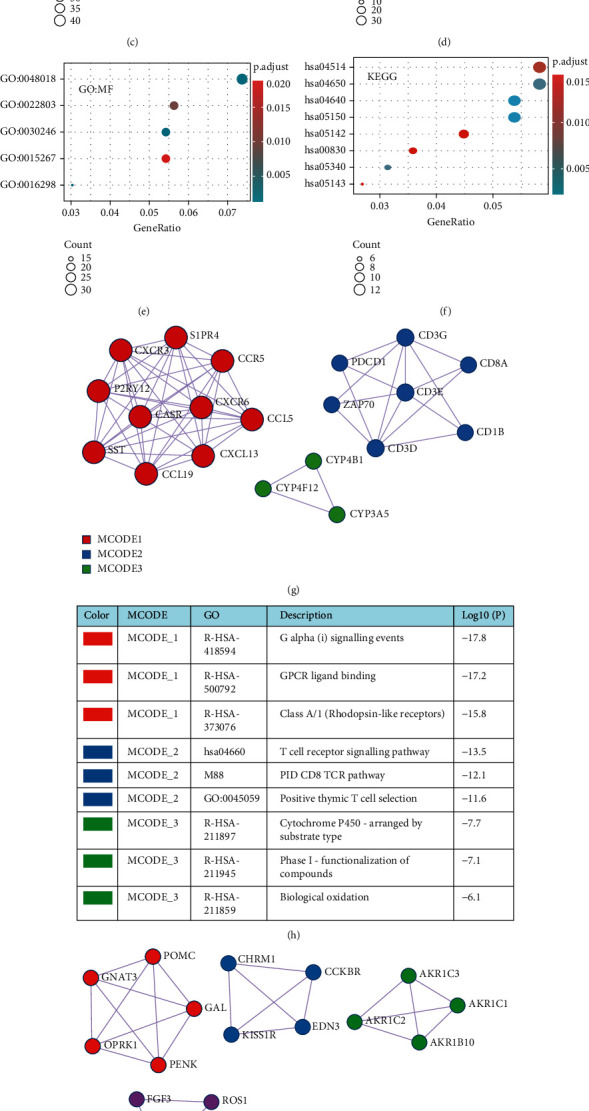
554 differentially expressed genes (DEGs) were identified between high-DHCR7 and low-DHCR7 expression cervical cancer patients. (a) Volcano plot of DEGs. (b) Heat map DEGs. (c–f) GO analysis and KEGG enrichment analysis of DEGs. (c) Enriched GO terms in the “Biological process” category. (d) Enriched GO terms in the “Cellular Component” category. (e) Enriched GO terms in the “Molecular Function” category. (f) Enriched KEGG pathways. (g and h) PPI network and MCODE components of downregulated DEGs. (i and j) PPI network and the MCODE components of upregulated DEGs. DEGs: differential expressed genes.

Using the cut-off threshold of adj. *P* < 0.05 and |log2FC| >1.0, 554 differentially expressed genes (DEGs) were identified between high-DHCR7 and low-DHCR7 cervical cancer patients. Among which, 261 genes were upregulated and 293 genes were downregulated (Figures 4(a) and 4(b)).

### 3.5. Functional Enrichment Analysis of DEGs

To estimate the functional significance of DHCR7 in cervical cancer, GO classification and KEGG enrichment analyses were conducted based on the 554 DEGs using clusterProfiler package. In the BP category, 148 enriched GO terms were identified. They were mainly enriched in T cell activation (GO: 0042110), regulation of T cell activation (GO: 0050863), regulation of lymphocyte activation (GO: 0051249), regulation of lymphocyte proliferation (GO: 0050670), and regulation of mononuclear cell proliferation (GO: 0032944) (Figure 4(c)). The results suggested there may be a link between aberrant DHCR7 expression and immunity. Results on the CC category revealed 18 enriched GO terms, including external side of plasma membrane (GO: 0009897), anchored component of membrane (GO: 0031225), intrinsic component of synaptic membrane (GO: 0099240), and integral component of synaptic membrane (GO: 0099699) (Figure 4(d)). MF category results showed 13 enriched GO terms associated with carbohydrate binding (GO: 0030246), receptor ligand activity (GO: 0048018), lipase activity (GO: 0016298), and channel activity (GO: 0015267) (Figure 4(e)). KEGG pathway analysis identified 15 enriched pathways, including *Staphylococcus aureus* infection (hsa05150), cell adhesion molecules (CAMs) (hsa04514), natural killer cell mediated cytotoxicity(hsa04650), and T cell receptor signaling pathway (hsa04660) (Figure 4(f)).

### 3.6. DHCR7 Related Signaling Pathways Based on GSEA

**Figure 5:**
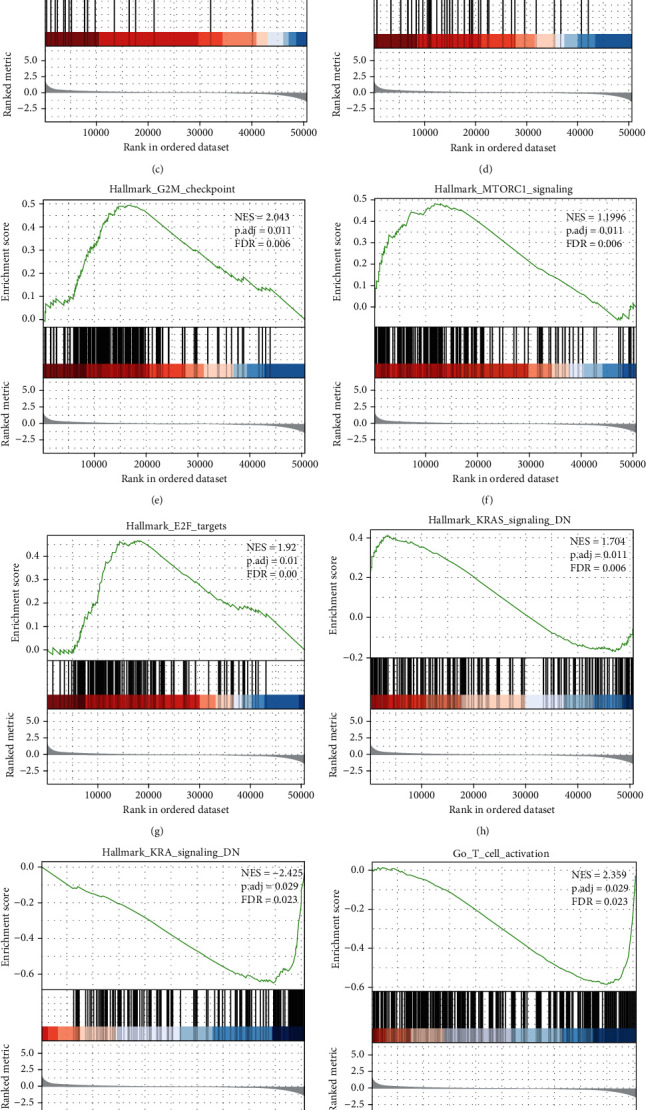
DHCR7-related signaling pathways obtained using GSEA. (a) Regulation of cholesterol biosynthetic process. (b) Fibroblast growth factor receptor signaling pathway. (c) WNT activated receptor activity. (d) PCG protein complex. (e) G2/M checkpoint. (f) mTORC1 signaling. (g) E2/F targets. (h) KRAS signaling. (i) T-cell receptor signaling pathway. (j) T cell activation. (k) Macrophage migration. (l) JAK/STAT signaling pathway.

GSEA was used to screen the potential biological processes and signaling pathway enriched using the 554 DEGs. This analysis revealed significant differences (FDR <0.05, adjusted *P*-value <0.05) in MSigDB Collection (c2.cp.v6.2.symbols).The most significantly enriched biological processes and signaling pathways were selected based on Normalized Enrichment Score (NES). DHCR7 was found to be correlated with WNT activated receptor activity, PCG protein complex, G2/M checkpoint, E2/F targets, mTORC1 signaling, KRAS signaling, regulation of cholesterol biosynthetic, fibroblast growth factor receptor signaling pathway, T-cell receptor signaling pathway, JAK/STAT signaling pathway, T cell activation, and macrophage migration (Figure 5).

### 3.7. The Correlation between DHCR7 Expression and Immune Infiltration

**Figure 6:**
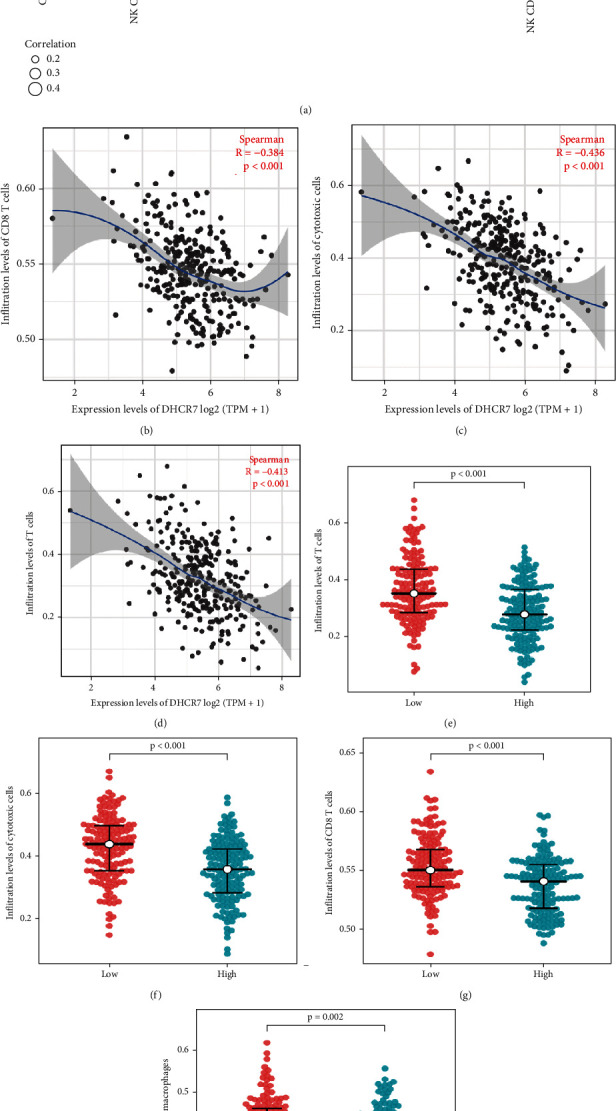
Correlation of DHCR7 expression with immune cell infiltration in cervical cancer. (a) Lollipop figure of correlation between DHCR7 and immune cell infiltration in cervical cancer. Correlation between DHCR7 expression and infiltration levels of (b) CD8+ T cells, (c) cytotoxic cells, and (d) T cells. (e–h) Immune cell infiltration in DHCR7-low and high expression patients. (e) T cells, (f) cytotoxic cells, (g) CD8+ T cells, and (h) macrophages.

To identify the correlation between immune infiltration levels of tumor-infiltrating lymphocytes (TILs) and DHCR7 expression in the cervical cancer, Spearman's correlation analysis was applied to 304 cervical cancer samples. Immune cell infiltration levels were quantified using ssGSEA. The result showed that DHCR7 expression was negatively correlated with cytotoxic cells, T cell, and CD8+T cell (*R* = -0.436, -0.413, and -0.384, respectively (*P* < 0.001, Figures 6(b)–6(d))). The Wilcoxon rank sum test indicated that infiltrating level of cytotoxic cells and T-cell was significantly higher in DHCR7-low samples in comparison with DHCR7-high ones (*P* < 0.001, Figures 6(e)–6(h)). These data showed that DHCR7 expression was correlated with reduced infiltration of cytotoxic cells, CD8+T cell, and T cell.

### 3.8. Protein-Protein Interaction (PPI) Network Construction and Hub Gene Selection

PPI analysis of cervical cancer DEGs was conducted using Metascape. The PPI network and MCODE components identified using the DEGs in the gene lists are shown on Figures 4(g)–4(j). The top 3 functional clusters of modules in the downregulated DEGs and 4 MCODE components in the upregulated DEGs detected from the PPI network by MCODE were involved in G alpha, T cell receptor, and CD8 TCR signaling pathways.

### 3.9. DHCR7 Expression Was Increased in Cervical Cancer Cell Lines and Downregulation of DHCR7 Suppressed Cell Proliferation

**Figure 7:**
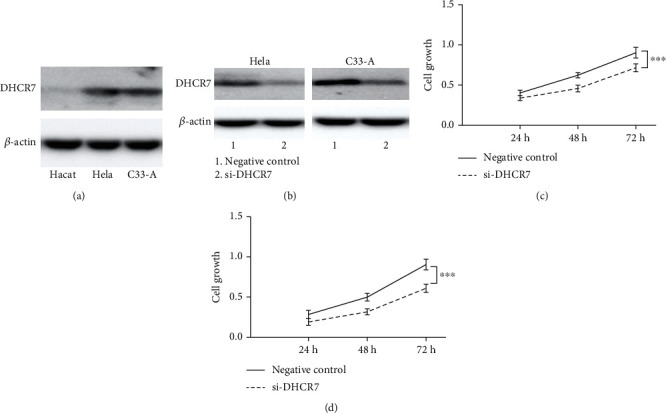
DHCR7 expression was increased in cervical cancer cell lines and downregulation of DHCR7 suppressed cell proliferation. (a) DHCR7 expression in non-tumor epithelial cell line (Hacat) and cervical cancer cell lines (Hela and C33-A). (b) DHCR7 expression was knocked down using siRNA in Hela and C33-A. Cell proliferation was significantly suppressed in DHCR7 low expression cells. (c) Hela. (d) C33-A. ^∗∗∗^, *P* < 0.001.

Western blot was performed to validate DHCR7 expression in cancer cell lines. Compared with non-tumor epithelial cells, DHCR7 expression level in Hela and C33-A cervical cancer cell lines was increased obviously (Figure 7(a)). We used siRNA of DHCR7 to knock down DHCR7 expression in Hela and C33-A (Figure 7(b)) and detected cell proliferation. After downregulation of DHCR7, cell proliferation was significantly suppressed in Hela and C33-A cells (*P* < 0.001; Figures 7(c) and 7(d)).

## 4. Discussion

DHCR7 (7-dehydrocholesterol reductase) is the terminal enzyme of the Kandutsch-Russell (MK–R) and Bloch pathways of cholesterol synthesis [22]. The MK–R pathway generates 7DHC for vitamin D3 synthesis [23]. DHCR7 and EBP serve as regulatory and catalytic subunits of antiestrogen binding site (AEBS), which is a target of tamoxifen [24]. DHCR7 inhibitors and 7-DHC are considered antiviral therapies against emerging or highly pathogenic viruses [25]. Gerrick et al. [26] found that DHCR7 was a key regulator to M2 macrophage polarization and suppresses IL-10 and TARC secretion. These findings highlight the cholesterol pathway as a potential target for macrophage reprogramming therapies.

We found that DHCR7 was differentially expressed between cancer and normal tissues. Elevated DHCR7 expression in cervical carcinoma was correlated with poor clinicopathological features including advanced clinical stage, advanced T stage, and lymph node invasion. ROC curve indicated that DHCR7 had a strong diagnostic power for cervical cancer. Patients with elevated DHCR7 levels had poor OS, DSS, and PFI. Though there were no OS or DSS differences between patients with different DHCR7 expression levels in adenosquamous subgroup, it should be noticed that only 52 patients were included in this subgroup. Multivariate analysis indicated that DHCR7 was an independent prognostic biomarker for clinical outcome. Increased DHCR7 predicted poor prognosis. Taken together, the current study highlighted the potential role of DHCR7 as a prognostic biomarker for cervical cancer.

For a more accurate prognosis prediction, nomograms combining DHCR7 with other clinical features were developed to predict the 1-, 3-, and 5-year of OS, PFI, and DSS in cervical cancer. This analysis revealed C-index values of 0.760, 0.703, and 0.803 for OS, PFI, and DSS, respectively (Figure S2), indicating reliable predictive performance.

It should be noticed that we also used the GEO cohort (GSE30760) to validate whether DHCR7 expression was associated with survival of cervical cancer. However, the result showed that survival of patients in the high DHCR7 and low DHCR7 expression groups was not significantly different (Figure S3). One reason may be that the sample size was very small. Only 48 patients were included in the cohort. Another reason may be that patient characteristic in GSE30760 was different with patient characteristics in TCGA database. There were 6.9% patients in TCGA database while no patients in GSE30760 were diagnosed with stage IV disease.

To investigate the potential biological function of DHCR7 and other co-expression genes, we performed functional enrichment and GSEA analysis. It was shown that DHCR7 was significantly involved in multiple signaling pathways, including G2/M checkpoint, mTORC1 signaling, KRAS signaling, WNT signaling, PCG protein complex, and regulation of cholesterol biosynthetic. The above pathways have also been previously implicated in other cancers. The results suggested that DHCR7 may promote tumor development and progression via these pathways in cervical cancer.

mTOR signaling plays critical roles in the tumor microenvironment (TME) and tumor angiogenesis [27]. Inhibition of tumor angiogenesis is an effective strategy for limiting tumor growth and preventing tumor metastasis [28]. As it is shown by other research, G2/M checkpoint abrogation prevents cancer cells from repairing DNA damage, especially radiation-induced DNA damage, which has emerged as an anticancer target [29]. In cancer cells, DNA damage is triggered by radioactive rays and chemical toxins and causes G2/M cell cycle arrest. Similarly, cancer cells become sensitive to radiotherapy after the abrogation of the radiation-induced G2 arrest [29, 30]. DHCR7 may modulate G2/M checkpoint in response to DNA damage. Interestingly, we found that DHCR7-high patients undergoing radiotherapy had worse prognosis. DHCR7 is a potential anticancer target to improve radiosensitivity and chemosensitivity in cervical cancer. However, this hypothesis needs experimental validation.

Immune infiltration and tumor microenvironment have emerged as modulators of cancer progression and may affect clinical outcomes. In this study, we used ssGSEA and Spearman's correlation analysis to unveil connections between DHCR7 expression and immune infiltration level. Our data demonstrated that DHCR7 was negatively correlated with the infiltration level of T cells, CD8+ T cells, and cytotoxic cells. Recent studies showed that the presence of tumor-infiltrating lymphocytes (TILs), particularly CD8+T cells, indicates favorable prognosis in several solid tumor types [31]. Patients with large T or B- cells infiltration or with T-cell or B-cell gene activation signature exhibit better survival [32, 33]. Additionally, primary tumor growth and metastasis are associated with decreased intratumoral immune T-cell densities [34]. High DHCR7 expression may suppress the function of immune system in cervical cancer by reducing CTLs and T-cell infiltration.

Biological progresses or pathways like T-cell receptor signaling, JAK/STAT, T cell activation, and macrophage migration were also detected in our study. T-cells are reported to have critical anti-tumor responses and have exhibited anticancer efficacy [35, 36]. Huang W et al. found that TCR-based immunotherapies may reduce solid tumor viability, including immune-checkpoint inhibitor refractory cancers [37]. Additionally, the PPI network analysis revealed that DHCR7 may participate in G alpha signaling events, T cell receptor signaling pathway, and the CD8 TCR pathway in cancer. Thus, we speculate that DHCR7 may affect cervical cancer development and progression by regulating the immune-related pathways.

Some limitations of our study should be noticed. Firstly, the study was based on bioinformatic analysis and without clinical validation. Secondly, it was a single-gene analysis. Thus, the analysis was not comprehensive. What's more, the size of control samples was small. Experimental studies using clinical specimens, in vitro and in vivo approaches are warranted.

## 5. Conclusion

In summary, we found that DHCR7 was significantly upregulated in cervical cancers and was correlated with unfavorable outcomes. DHCR7 may be a potential biomarker for poor survival in cervical cancer.

## Figures and Tables

**Table 1:** Patients' characteristics in the TCGA cohort.

Characters	Overall, *n* (%)
Age (y) (median [IQR])	
<=50	185 (60.8%)
>50	119 (39.2%)
Primary tumor (T)	
T1	140 (46.0%)
T2	71 (23.4%)
T3	20 (6.6%)
T4	10 (3.3%)
Unknown	63 (20.7%)
Lymph node status (N)	
N0	133 (43.8%)
N1	60 (19.7%)
Unknown	111 (36.5%)
Metastasis (M)	
M0	116 (38.2%)
M1	10 (3.3%)
Unknown	178 (58.5%)
Radiation therapy	
No	122 (40.1%)
Yes	182 (59.9%)
Primary therapy outcome	
CR	181 (59.5%)
PD	22 (7.2%)
PR	8 (2.6%)
SD	6 (2.0%)
Unknown	87 (28.6%)
Histological type	
Adenosquamous	52 (17.1%)
Squamous cell carcinoma	252 (82.9%)
Histologic grade	
G1	18 (5.9%)
G2	135 (44.4%)
G3	118 (38.8%)
G4	1 (0.3%)
Unknown	32 (10.5%)
Clinical stage	
Stage I	162 (53.2%)
Stage II	69 (22.6%)
Stage III	45 (14.8%)
Stage IV	21 (6.9%)
Unknown	7 (2.3%)
Menopause status	
Peri	25 (8.2%)
Post	82 (27.0%)
Pre	124 (40.8%)
Unknown	73 (24.0%)
Birth control pill history	
No	89 (56.7%)
Yes	68 (43.3%)
Keratinizing squamous cell carcinoma present	
No	119 (39.1%)
Yes	185 (60.9%)
Smoker	
No	144 (47.4%)
Yes	117 (38.5%)
Unknown	43 (14.1%)

**Table 2:** Logistic regression analysis of correlation of DHCR7 expression with clinicopathological factors.

Characteristics	Numbers	Odds ratio (OR)	*P* value
T stage (T2 and T3 and T4 vs. T1)	241	1.01 (1.00-1.02)	0.028
N stage (N1 vs. N0)	193	1.01 (1.00-1.02)	0.043
M stage (M1 vs. M0)	126	1.01 (1.00-1.03)	0.049
Clinical stage (stage II and stage III and stage IV vs. stage I)	297	1.01 (1.00-1.01)	0.026
Primary therapy outcome (CR vs. PD and SD and PR)	217	0.99 (0.98-1.00)	0.012
Histological type (squamous cell carcinoma vs. adenosquamous)	304	1.00 (0.99-1.01)	0.452
Histologic grade (G3 and G4 vs. G1 and G2)	272	1.00 (1.00-1.01)	0.521
Keratinizing squamous cell carcinoma present (yes vs. no)	304	1.00 (1.00-1.01)	0.630
PIK3CA status (Mut vs. WT)	286	1.00 (0.99-1.01)	0.593

**Table 3:** Associations with overall survival (OS), progression-free interval (PFI), disease-specific survival (DSS), and clinicopathologic characteristics in TCGA patients (a) Cox regression. (b) Multivariate survival model after variable selection.

Characteristics	Total (*N*)	OS		PFI		DSS	
		HR (95% CI)	*P* value	HR (95% CI)	*P* value	HR (95% CI)	*P* value
(a) Cox regression							
T stage (T2 and T3 and T4 vs. T1)	241	1.846 (1.045-3.260)	**0.035**	1.656 (0.976-2.812)	0.062	2.047 (1.068-3.926)	**0.031**
N stage (N1 vs. N0)	193	2.695 (1.358-5.349)	**0.005**	1.983 (0.986-3.990)	0.055	3.303 (1.446-7.541)	**0.005**
Clinical stage (stage II and stage III and stage IV vs. stage I)	297	1.429 (0.896-2.280)	0.134	1.308 (0.821-2.084)	0.258	1.608 (0.941-2.749)	0.082
Primary therapy outcome (CR vs. PD and SD and PR)	217	0.074 (0.040-0.138)	**<0.001**	0.126 (0.074-0.216)	**<0.001**	0.059 (0.030-0.116)	**<0.001**
Radiation therapy (yes vs. no)	304	1.153 (0.681-1.951)	0.596	1.288 (0.754-2.200)	0.354	1.772 (0.891-3.522)	0.103
Histological type (squamous cell carcinoma vs. adenosquamous)	304	1.010 (0.530-1.926)	0.976	0.778 (0.433-1.397)	0.400	0.956 (0.467-1.958)	0.901
Menopause status (post vs. pre and peri)	231	1.275 (0.744-2.185)	0.376	1.091 (0.640-1.861)	0.749	1.233 (0.678-2.243)	0.493
Histologic grade (G3 and G4 vs. G1 and G2)	272	0.889 (0.527-1.502)	0.661	1.594 (0.967-2.627)	0.067	0.955 (0.530-1.720)	0.877
Smoker (yes vs. no)	261	1.470 (0.900-2.401)	0.124	0.998 (0.613-1.623)	0.993	1.309 (0.747-2.295)	0.347
Birth control pill history (yes vs. no)	157	0.677 (0.326-1.404)	0.294	0.938 (0.495-1.776)	0.844	0.705 (0.314-1.581)	0.396
Keratinizing squamous cell carcinoma present (yes vs. no)	304	1.395 (0.813-2.394)	0.227	1.077 (0.657-1.765)	0.769	1.789 (0.938-3.412)	0.077
Age (>50 vs. <=50)	304	1.317 (0.825-2.101)	0.248	1.612 (1.012-2.568)	**0.044**	1.333 (0.780-2.278)	0.292
Height (>160 vs. <=160)	261	1.092 (0.633-1.883)	0.752	0.830 (0.489-1.410)	0.491	0.915 (0.498-1.683)	0.776
Weight (>70 vs. <=70)	275	0.736 (0.446-1.214)	0.230	0.645 (0.390-1.066)	0.087	0.802 (0.453-1.420)	0.450
Race (Asian and Black or African American vs. White)	259	0.841 (0.427-1.658)	0.618	1.066 (0.553-2.057)	0.848	0.770 (0.344-1.724)	0.526
PIK3CA status (Mut vs. WT)	286	1.011 (0.599-1.707)	0.967	0.994 (0.589-1.679)	0.983	1.097 (0.606-1.989)	0.759
DHCR7 (high vs. low)	304	1.758 (1.088-2.841)	**0.021**	2.224 (1.352-3.657)	**0.002**	2.258 (1.271-4.013)	**0.005**

(b) Multivariate analysis							
T stage (T2 and T3 and T4 vs. T1)	241	0.812 (0.312-2.111)	0.669			0.947 (0.229-3.918)	0.940
N stage (N1 vs. N0)	193	2.670 (1.094-6.515)	**0.031**			2.605 (0.998-6.801)	**0.050**
Primary therapy outcome (CR vs. PD and SD and PR)	217	0.185 (0.068-0.506)	**0.001**	0.151 (0.056-0.411)	**<0.001**	0.126 (0.042-0.381)	**<0.001**
Age (>50 vs. <=50)	304			0.543 (0.216-1.363)	0.193		
DHCR7 (high vs. low)	304	2.973 (1.177-7.510)	**0.021**	2.517 (1.082-5.856)	**0.032**	3.207 (1.194-8.611)	**0.021**

## Data Availability

The datasets used and/or analyzed during the current study are available from the corresponding authors on reasonable request.

## Supplementary Materials

Supplementary 1 Figure S1 showed the Kaplan-Meier survival curves of patients with high and low DHCR7 expression in the adenosquamous cervical cancer subgroup.

Supplementary 2 Figure S2 showed the nomogram and calibration curves of DHCR7.

Supplementary 3 Figure S3. The GEO cohort (GSE30760) was used to validate whether DHCR7 expression was associated with survival of cervical cancer. However, the result showed that survival of patients in the high DHCR7 and low DHCR7 expression groups was not significantly different.
