# Supplementary figures and images for: High DHCR7 Expression Predicts Poor Prognosis for Cervical Cancer
Source: Comput Math Methods Med. 2022 Sep 16;2022:8383885. doi: 10.1155/2022/8383885 (PMC9508458; doi:10.1155/2022/8383885)

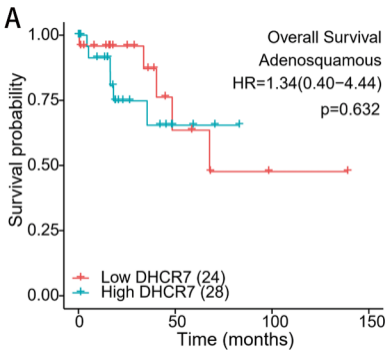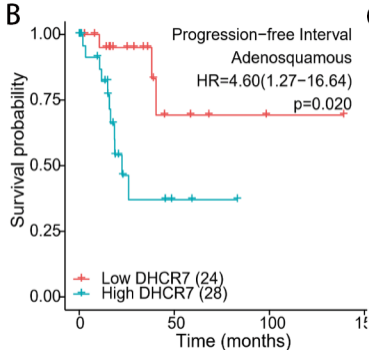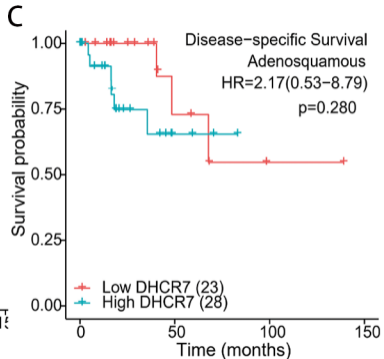

Supplement: Supplementary 1 — Figure S1 showed the Kaplan-Meier survival curves of patients with high and low DHCR7 expression in the adenosquamous cervical cancer subgroup. [file 8383885.f1.pdf]

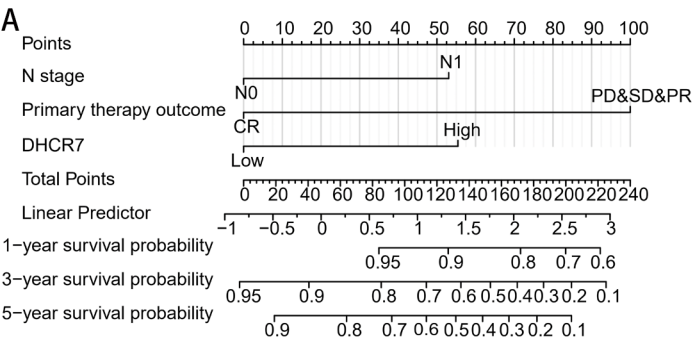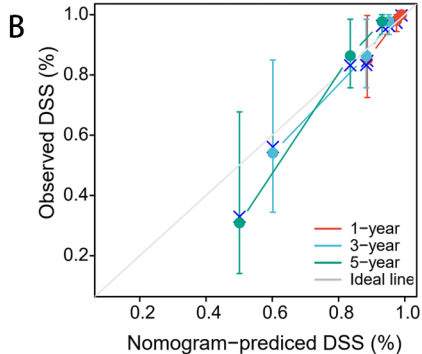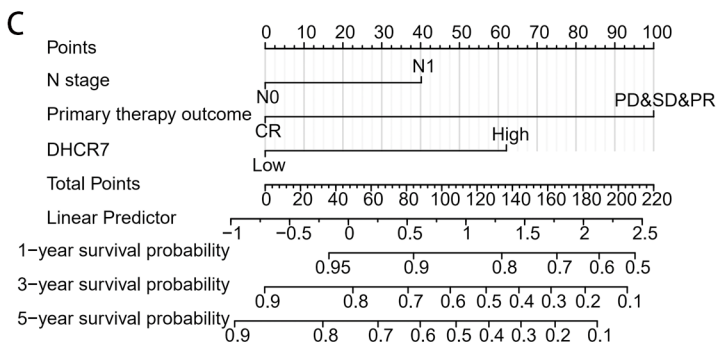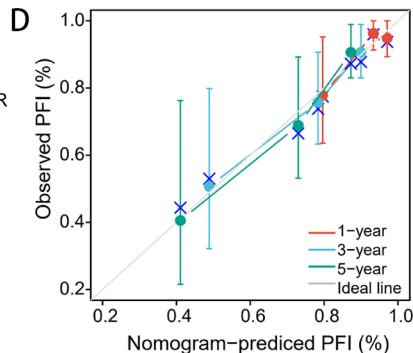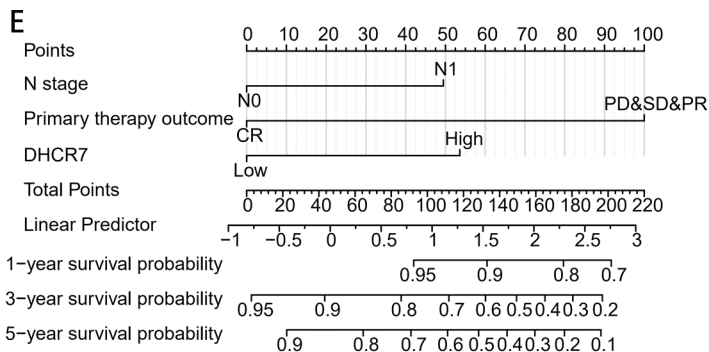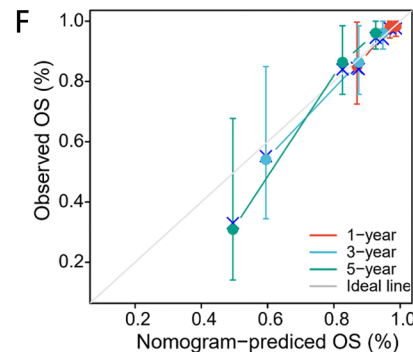

Supplement: Supplementary 2 — Figure S2 showed the nomogram and calibration curves of DHCR7. [file 8383885.f2.pdf]

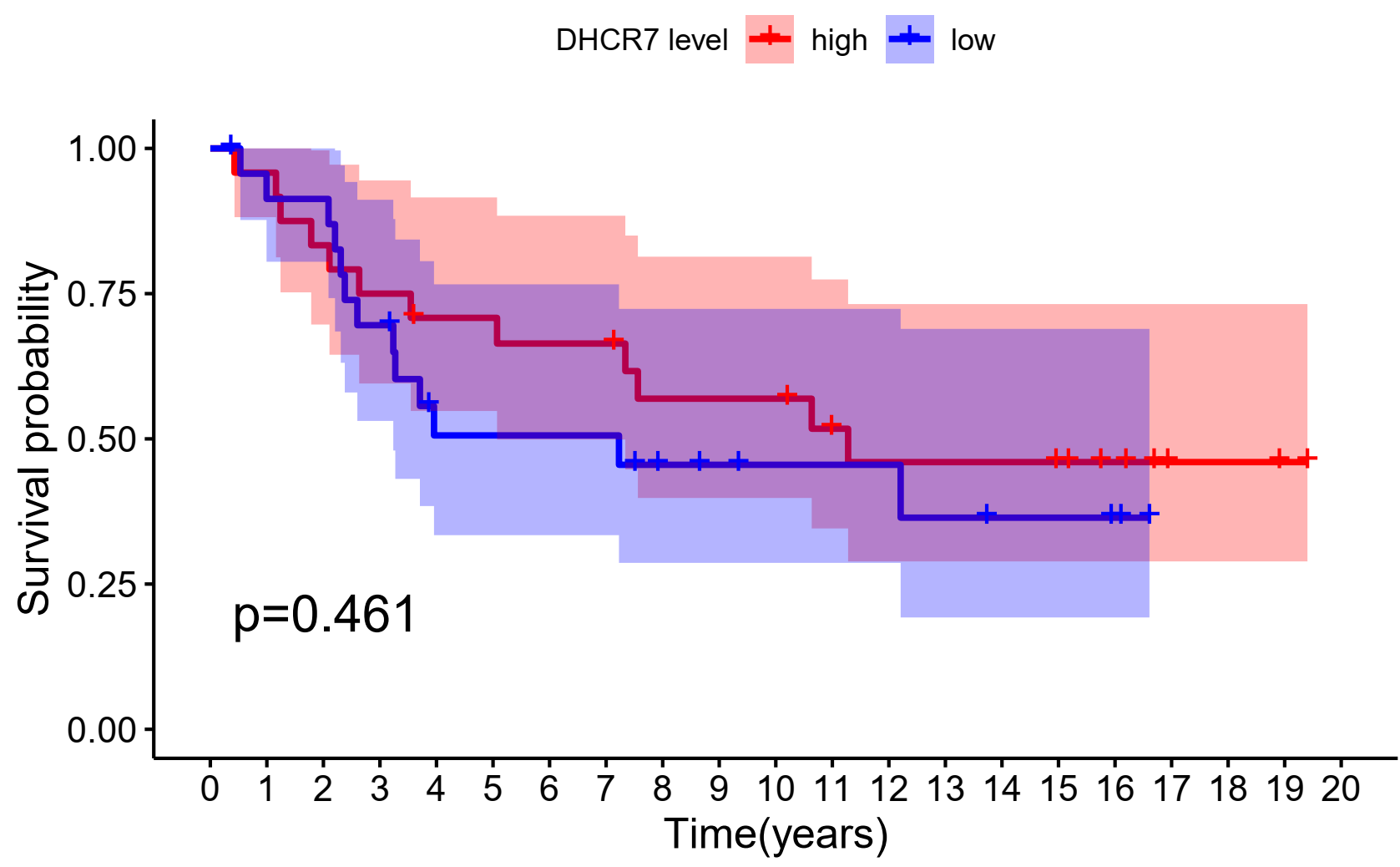

Supplement: Supplementary 3 — Figure S3. The GEO cohort (GSE30760) was used to validate whether DHCR7 expression was associated with survival of cervical cancer. However, the result showed that survival of patients in the high DHCR7 and low DHCR7 expression groups was not significantly different. [file 8383885.f3.tif]
